# Stable Differences in Intrinsic Mitochondrial Membrane Potential of Tumor Cell Subpopulations Reflect Phenotypic Heterogeneity

**DOI:** 10.1155/2011/978583

**Published:** 2011-07-02

**Authors:** Michele A. Houston, Leonard H. Augenlicht, Barbara G. Heerdt

**Affiliations:** Department of Oncology, Albert Einstein Cancer Center, Montefiore Medical Center, 111 East 210th Street, Bronx, NY 10467, USA

## Abstract

Heterogeneity among cells that constitute a solid tumor is important in determining disease progression. Our previous work established that, within a population of metastatic colonic tumor cells, there are minor subpopulations of cells with stable differences in their intrinsic mitochondrial membrane potential (ΔΨm), and that these differences in ΔΨm are linked to tumorigenic phenotype. Here we expanded this work to investigate primary mammary, as well as colonic, tumor cell lines. We show that within a primary mammary tumor cell population, and in both primary and metastatic colonic tumor cell populations, there are subpopulations of cells with significant stable variations in intrinsic ΔΨm. In each of these 3 tumor cell populations, cells with relatively higher intrinsic ΔΨm exhibit phenotypic properties consistent with promotion of tumor cell survival and expansion. However, additional properties associated with invasive potential appear in cells with higher intrinsic ΔΨm only from the metastatic colonic tumor cell line. Thus, it is likely that differences in the intrinsic ΔΨm among cells that constitute primary mammary tumor populations, as well as primary and metastatic colonic tumor populations, are markers of an acquired tumor phenotype which, within the context of the tumor, influence the probability that particular cells will contribute to disease progression.

## 1. Introduction

Cancer is a highly heterogeneous disease and diversity among cells that make up solid tumors likely influences the probability of tumor expansion and progression [1]. Abnormalities in mitochondrial (mt) structure and function [2–4] and a general elevation in the mitochondrial membrane potential (ΔΨm) [5–9] have been linked to malignant transformation. We previously reported that within the population of SW620 cells, a cell line established from a human metastatic colon tumor [10], there are minor subpopulations of cells with significant stable differences in intrinsic ΔΨm and that these differences in ΔΨm are linked to tumorigenic phenotype [11].

To address the question of whether stable differences in intrinsic ΔΨm exist among cells in other tumor cell populations and, if so, whether these differences are similarly linked to tumorigenic phenotypes, we generated single cell subclones from SW480 cells, a cell line established from the primary colon tumor of the same patient from which SW620 cells were established [10] and from MCF7 primary mammary carcinoma cells [12].

Here we report that, consistent with our previous work, there are significant and stable differences in the intrinsic ΔΨm among a minor fraction of cells within a population of both SW480 and MCF7 cells and that these differences in ΔΨm are again linked to tumorigenic phenotype, with elevations in the intrinsic ΔΨm associated with decreased sensitivity to the chemoprotective agent butyrate and increased constitutive, hypoxia-independent VEGF secretion. Moreover, we demonstrate that unlike the differences in ΔΨm of clones from SW620 cells, the intrinsic ΔΨm of subclones derived from the SW480 primary colonic tumor cell population, or from the MCF7 primary mammary tumor cell population, do not impact invasive potential.

Thus, our data establish significant stable heterogeneity in the intrinsic ΔΨm among subpopulations of cells of both a primary and metastatic colon tumor and from a primary mammary tumor and suggest that differences in intrinsic ΔΨm are not random variations or passive indices of heterogeneity, but rather markers of acquired behavior of subpopulations cells which, within the context of the tumor, are likely selected for, and contribute to, clinical progression of the disease [13].

## 2. Materials and Methods

### 2.1. Quantitation of ΔΨm (Mitochondrial Membrane Potential)

The ΔΨm was determined as previously described by staining cells with the ΔΨm-dependent fluorescent dye JC-1 (5,5′,6,6′-tetrachloro-1,1′,3,3′ tetraethylbenzimidazol carbocyanineiodide; Molecular Probes, Eugene, OR) and analyzing fluorescence emission by flow cytometry in detection channel 2 (FL-2) [14–17].

### 2.2. Quantitation of Viability in CoCl_2_ Simulated Hypoxia

Cells grown to 80% confluence were incubated for 24 hours in medium alone or in medium containing 100 mM CoCl_2_. Cell viability was then determined by the MTT assay [18].

### 2.3. Quantitation of Response to Butyrate

Cells at approximately 80% confluence were treated with medium alone or with medium containing 5 mM sodium butyrate (NaB) (Sigma, St. Louis, MO). Seventy-two hours later, cell viability was determined by the MTT assay [18].

### 2.4. Quantitation of Secretion of Matrilysin (MMP7) and Vascular Endothelial Growth Factor (VEGF)

Cells were seeded into 96-well plates and grown to approximately 80% confluence. Conditioned tissue culture medium was then harvested, and MMP7 and VEGF_165_ protein levels were quantified by ELISA (R&D Systems, Inc., Minneapolis, MN) according to the manufacturer's protocol. Constitutive MMP7 and VEGF_165_ secretion levels were normalized by determining cells per well by the MTT assay and expressed relative to the population of cells.

### 2.5. Quantitation of Fascin Protein by Immunoblotting

Cells grown to approximately 80% confluence were washed twice in ice cold PBS and lysed in buffer containing 0.5% NP-40, 1% Triton X-100, 0.2 mM sodium orthovanadate, 0.2 mM PMSF, 1 mM EDTA (pH 8), 1 mM EGTA (pH 8), 150 mM NaCl, and 10 mM Tris pH 7.4. Lysates were cleared by centrifugation and protein concentration determined (Bio-Rad, Hercules, CA). Thirty *μ*g of cell lysates were size-fractionated on 4–20% acrylamide SDS-PAGE gels (Bio-Rad, Hercules, CA) and blotted onto PVDF membranes (Amersham, Arlington Heights, IL). Replicate blots were probed with antibodies directed against fascin (Millipore, Billerica, MA) or actin (EMD Biosciences, Gibbstown, NJ), followed by incubation with appropriate secondary antibodies. Reactions were detected by enhanced chemiluminescence and quantified by densitometry using Kodak IS4000R and Kodak Molecular Imaging Software.

### 2.6. Quantitation of Cell Invasion

Cells were seeded into chambers consisting of a reconstituted basement membrane supported by an underlying polycarbonate membrane (Millipore, Billerica, MA). Invasion through the basement membrane was determined by the staining and subsequent quantification by optical density at 560 nM, of cells adhering to the polycarbonate membrane.

### 2.7. Statistical Analyses

Data from at least 3 independent determinations of the intrinsic ΔΨm were analyzed by Bonferroni's Multiple Comparison Test. Mean data from other determinations were evaluated as a function of the relative intrinsic ΔΨm using linear regression analyses.

## 3. Results

### 3.1. Subpopulations of Cells within Primary Colonic and Mammary Carcinoma Cell Populations Exhibit Stable Differences in Intrinsic ΔΨm

JC-1 is a lipophilic, cationic fluorescent dye that exhibits ΔΨm-dependent uptake, accumulation, and aggregate formation in the mitochondria [19]. The emission intensity of these aggregates at 590 nm, analyzed by flow cytometry in fluorescence detection channel 2 (FL-2), is a sensitive index of the cell's ΔΨm [15, 17]. In our previous work, we demonstrated that the distribution of JC-1 staining of metastatic colonic tumor SW620 cells reflected the presence of subpopulation of cells with stable differences in intrinsic ΔΨm [11]. 

**Figure 1:**
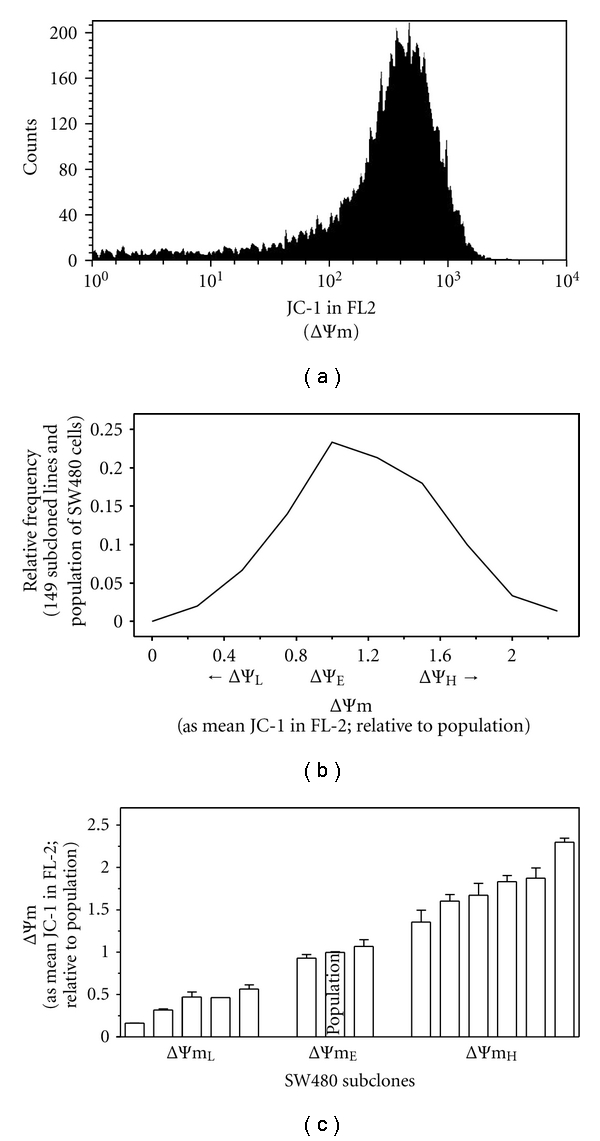
Subpopulations of cells within a primary colonic carcinoma cell population exhibit stable differences in intrinsic ΔΨm. (a) The SW480 cell line stained with JC-1 and analyzed by flow cytometry in FL-2. (b) The parental population of SW480 and 149 subcloned cell lines were stained with JC-1, analyzed by flow cytometry, the intrinsic ΔΨm each subcloned line was expressed relative to that of the unselected population of SW480 cells and plotted as a frequency distribution. (c) 13 subcloned cell lines derived from SW480 cells were selected from further study: the intrinsic ΔΨm of subclones “ΔΨm_L_” range from 0.17 and 0.50 fold lower; subclones “ΔΨm_E_” equivalent; and subclones “ΔΨm_H_” from 1.60 to 2.30 fold higher than that of the population of SW480 cells (also see Figure 1(b)). Mean and SEM of multiple ΔΨm determinations made over approximately 1 year. The intrinsic ΔΨm of all of the ΔΨm_L_ subclones are statistically different from all of the ΔΨm_H_ subclones (Bonferroni's Multiple Comparison Test; *P* ≤ 0.05).

**Figure 2:**
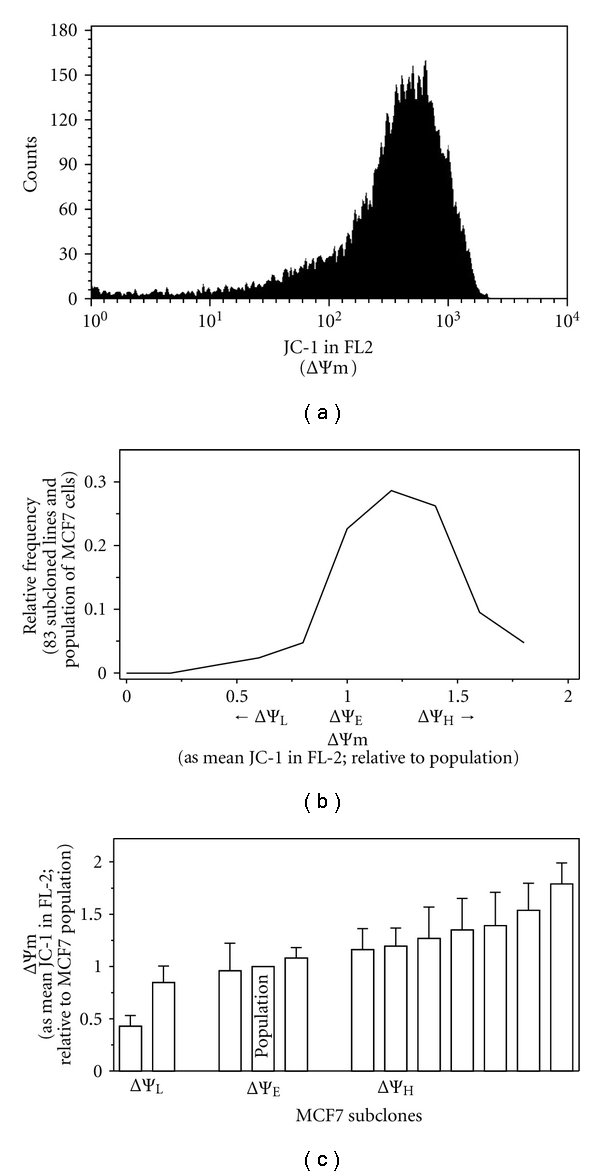
Subpopulations of cells within primary mammary carcinoma cell population exhibit stable differences in intrinsic ΔΨm. (a) The MCF7 cell line stained with jc-1 and analyzed by flow cytometry in FL-2. (b) The parental population of MCF7 and 83 subcloned cell lines were stained with JC-1, analyzed by flow cytometry, the Intrinsic ΔΨm each subcloned line was expressed relative to that of the unselected population of MCF7 cells and plotted as a frequency distribution. (c) 11 subcloned cell lines derived from SW480 cells were selected from further study. Mean and SEM of triplicate ΔΨm determinations made over approximately 3 months. Lower intrinsic ΔΨm are statistically different from higher intrinsic ΔΨm (Bonferroni's Multiple Comparison Test; *P* ≤ 0.05).

Here we investigated whether the distribution of JC-1 stained SW480 cells (Figure 1(a)), a cell line established from the primary colon tumor of the same patient from which metastatic SW620 cells were established [10], and from MCF7 cells (Figure 2(a)), a primary mammary tumor [12], also reflected stable differences in intrinsic ΔΨm among cells that constitute the population.

Similar to our previous work [11], suspensions of SW480 or MCF7 cells were diluted such that there was a high probability that a single cell was placed into standard tissue culture wells. From 272 wells seeded with SW480 cells, 149 subclones were generated and expanded. Assuming that a single cell was placed into each well, these data suggest that 54.8% of primary colonic tumor SW480 cells were capable of clonal growth and expansion, comparable to our previous work in which 53.8% of metastatic colonic tumor SW620 cells were capable of generating subcloned cell lines [11]. 

The SW480 parental population and each of 149 derived subcloned cell lines were then stained with JC-1 and analyzed by flow cytometry. The intrinsic ΔΨm of each of subcloned lines was expressed relative to that of the unselected population of cells and plotted as a frequency distribution (Figure 1(b)). The relative ΔΨm of the 149-subcloned cell lines derived from SW480 cells ranged from approximately 0.17-fold below, to 2.30-fold above, that of the mean of the population.

For subsequent investigations, we focused on 13 of the SW480 subcloned lines falling into 3 groups. As shown in Figures 1(b) and 1(c), the mitochondrial membrane potentials of subcloned lines in the group designated ΔΨm_L_ are between 0.17- and 0.50-fold lower than that of the population of SW480 cells; the ΔΨm of the subcloned lines in the ΔΨm_E_ subgroup are comparable to that of the population of cells; and the ΔΨm of the subclones in the ΔΨm_H_ subgroup are 1.60- to 2.30-fold higher than that of the population of cells. 

Similar to our previous work investigating intrinsic ΔΨm of SW620-derived clones [11, 20], the intrinsic ΔΨm of subcloned cell lines derived from SW480 cell are highly stable, demonstrated by the consistency among multiple determinations made over a period of approximately 1 year and hence standard errors that are consistently <10% of the means (Figure 1(c)). Moreover, evaluation of these data by Bonferroni's Multiple Comparison Test shows that the intrinsic ΔΨm of all of the ΔΨm_L_ subclones are statistically different from all of the ΔΨm_H_ subclones (*P* ≤ 0.05). 

Similarly, 83 subclones derived from the MCF7 population were expanded, the intrinsic ΔΨm of each was determined by JC-1, expressed relative to the population and plotted as a frequency distribution (Figure 2(b)). The relative ΔΨm of these subcloned cell lines ranged from approximately 0.43-fold below to 1.80-fold above that of the mean of the population. 

We focused on 11 of the MCF7 subcloned lines; 2 with intrinsic ΔΨm at least 15% lower, 2 with ΔΨm comparable, and 7 with intrinsic ΔΨm at least 15% higher than that of the population of MCF7 cells (Figure 2(c)). Bonferroni's Multiple Comparison Test of these data shows that the lower intrinsic ΔΨm are statistically different from the higher intrinsic ΔΨm (*P* ≤ 0.05). 

Thus, these data extend the finding of minor subpopulations of cells with significant stable differences in intrinsic ΔΨm from SW620 metastatic colon tumor cells [11] to the primary colon tumor cells of the same patient and to primary MCF7 mammary carcinoma cells.

### 3.2. Differences in the Intrinsic ΔΨm of Subcloned Cell Lines Derived from Primary Colon and Mammary Tumors Are Linked to Sensitivity to the Chemoprotective Agent Sodium Butyrate (NaB)

The unbranched short chain fatty acid butyrate is a natural constituent of the colonic contents, present at high concentrations due to its generation during fiber fermentation in the large intestine [21, 22]. Rapidly taken up by cells, butyrate enters the mitochondria where it undergoes *β*-oxidation [23]. In addition to functioning as the primary energy source for colonic epithelial cells [24], NaB mediates maturation and apoptotic pathways *in vitro* and *in vivo* [25–27] thereby likely suppressing development and/or progression of colon cancer [28–31]. 

Butyrate also has potent effects on a variety of other cell types, including normal and malignant mammary epithelial cells [32, 33]. However, due to its rapid* in vivo* metabolism, it is difficult to achieve and maintain effective serum levels even when butyrate salts are administered by continuous i.v. [34, 35]. We have reported that tributyrin, a triglyceride analogue of butyric acid that generates and maintains higher serum butyrate levels than NaB [36, 37], initiates growth arrest and apoptosis of MCF7 cells associated with mitochondrial activity [16] and found that dietary tributyrin effectively decreases the incidence, and increases the latency, of carcinogen induced mammary tumors in BALB/c mice (Heerdt et al., unpublished observation). 

**Figure 3:**
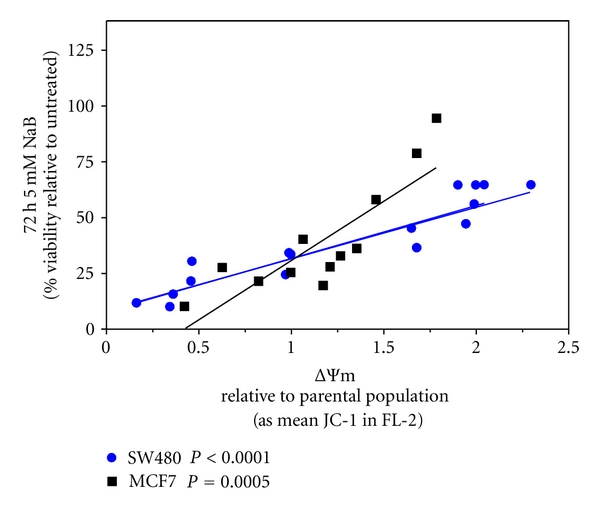
Differences in the Intrinsic ΔΨm of subcloned cell lines derived from primary colon and mammary tumors and from a metastatic colon tumor are linked to sensitivity to the chemoprotective agent sodium butyrate (NaB). The SW480 and MCF7 cell lines, and subclones derived from each of the cell lines, were exposed to 5 mm NaB for 72 hours. Viability was determined by MTT assay, expressed relative to untreated cells, and plotted as a function of relative ΔΨm.

We have shown that the ΔΨm plays a critical role in NaB initiated cell cycle arrest and apoptotic pathways in SW260 cells [14, 17] and that differences in the intrinsic ΔΨm significantly impact cellular sensitivity to NaB [11, 14, 20]. Therefore, to investigate the relationship between the intrinsic ΔΨm of primary tumor SW480 and MCF7 derived subclones and NaB sensitivity, the population of SW480- and the 13-derived subcloned cell lines (Figure 1(c)), and the MCF7 population and 11 derived subclones (Figure 2(c)), were exposed to 5 mM NaB, a physiologically relevant concentration [21, 38], for 72 hours. Viability relative to untreated cells was then determined and plotted as a function of relative ΔΨm. As shown in Figure 3, differences in the cellular sensitivity to NaB are a function of the intrinsic ΔΨm of subcloned cell lines derived from SW480 cells (*P* < 0.0001) or from MCF7 cells (*P* = 0.0005) with elevated ΔΨm linked to decreased sensitivity, consistent with our previous work [11, 14, 20]. 

### 3.3. Differences in the Intrinsic ΔΨm of Subcloned Cell Lines Derived from Primary Colonic and Mammary Tumors Are Linked to Phenotypes Associated with Solid Tumor Expansion

**Figure 4:**
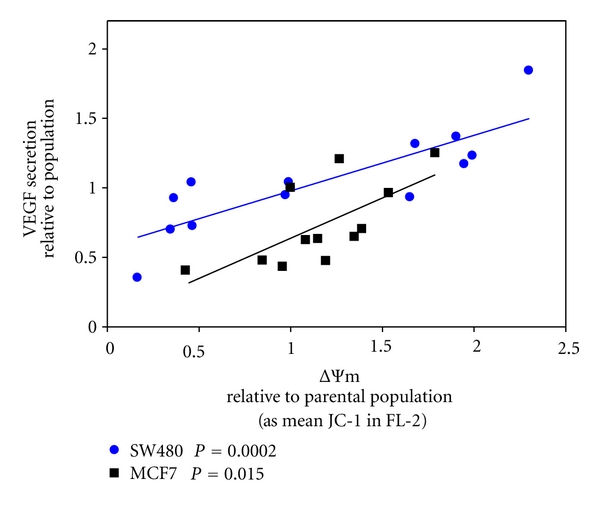
Differences in the intrinsic ΔΨm of subcloned cell lines derived from primary colonic and mammary tumors are linked to phenotypes associated with solid tumor expansion. VEGF was quantified by ELISA in condition medium harvest from SW480 and MCF7 cell lines, and subclones derived from each of the cell lines, and normalized for cells/well by the MTT assay. VEGF secretion levels are plotted as a function of relative ΔΨm.

Rapid growth and expansion of solid tumors produces regions where the demand for oxygen exceeds that which can be obtained by diffusion from existing blood vessels, resulting in areas of hypoxia. Therefore, to expand beyond approximately 1-2 mm in diameter, tumors need to acquire an independent blood supply [39–41]. Vascular endothelial growth factor (VEGF) promotes new endothelial and lymphatic vessel formation in tumors and has been linked to poor prognosis [42]. Our previous work has established a significant impact of the intrinsic ΔΨm of clones derived from SW620 cells and constitutive, hypoxia-independent secretion of VEGF [11, 20]. Here we show that, similarly, the intrinsic ΔΨm of SW480 and MCF7 subclones is linked to VEGF secretion (*P* = 0.0002 and *P* = 0.015, resp.) with elevated ΔΨm generally associated with increased levels of secretion (Figure 4). 

### 3.4. Differences in the Intrinsic ΔΨm of Subcloned Cell Lines Derived from a Metastatic Colonic Tumor, but Not from the Paired Primary Colonic Tumor or from a Primary Mammary Tumor, Are Linked to Phenotypic Markers Associated with Invasion

For successful invasion and metastasis, tumor cells must degrade and penetrate the extracellular matrix. Key enzymes in these processes include matrix metalloproteinases (MMPs). Whereas most MMPs are produced by stromal cells, MMP7 (matrilysin) is synthesized by tumor cells and its elevated expression, particularly at a tumor's invasive edge, is characteristic of metastatic cancer cells [43–45]. We have previously shown that metastatic SW620 cells constitutively secrete MMP7 and that secretion levels are a function of the intrinsic ΔΨm, with elevated intrinsic ΔΨm associated with higher MMP7 secretion [11].

**Figure 5:**
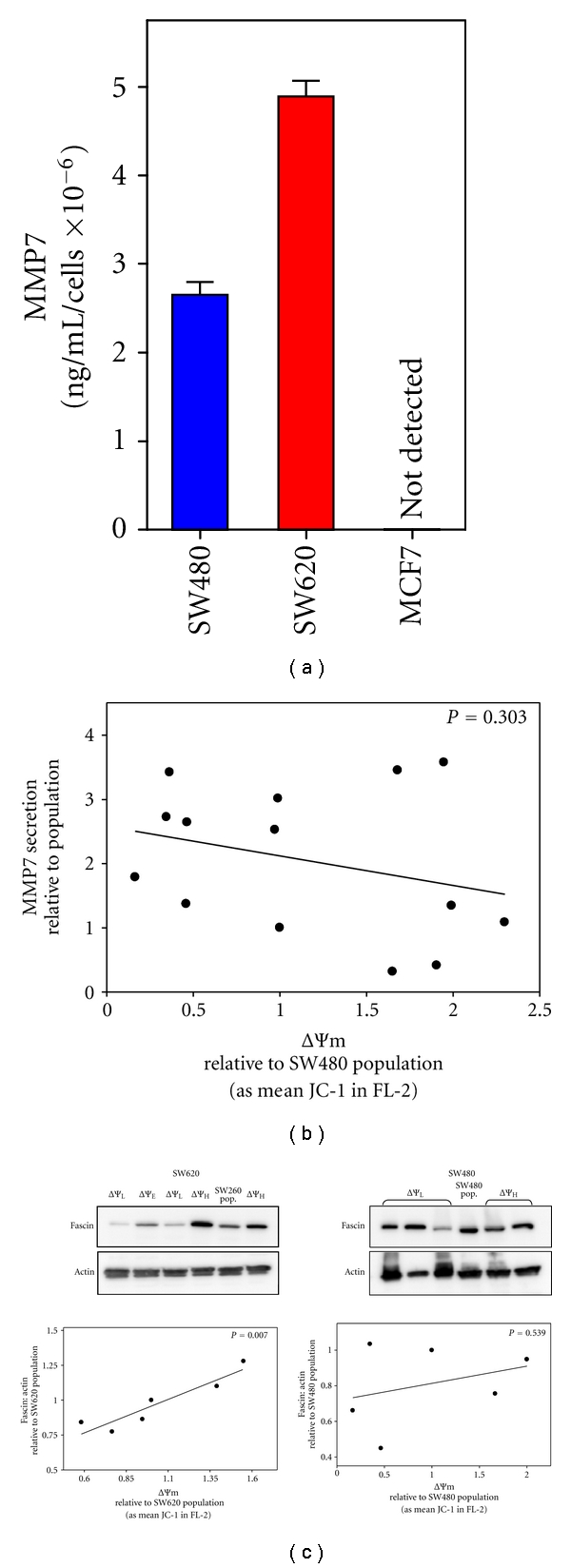
Differences in the intrinsic ΔΨm of subcloned cell lines derived from a metastatic colonic tumor, *but not from the paired primary colon tumor*, are linked to phenotypic markers associated with invasion. (a) and (b) MMP7 secretion levels were quantified by ELISA in condition medium harvest from SW480 and SW620 Cells (a) and from 13 subcloned cell lines derived from SW480 cells (b) and normalized for cells/well by the MTT assay. Secretion levels in parental SW480 and derived subclones are plotted as a function of relative ΔΨm (b). (c) Fascin levels were determined in the SW480 and SW620 cell lines, and subclones derived from each of the cell lines, by immunoblotting normalized to actin. Reactions were quantified by densitometry and plotted as a function of relative ΔΨm.

To investigate whether there is a similar relationship between constitutive MMP7 secretion and the intrinsic ΔΨm of SW480- or MCF7-derived subclones, MMP7 protein levels were quantified in harvested conditioned medium and plotted as a function of the ΔΨm of each cell line. Consistent with previous reports, we found that mean constitutive MMP7 secretion in the parental population of SW480 cells is approximately 50% lower than that of the metastatic SW620 cell population (Figure 5(a); *P* < 0.0001) [46, 47], and that MMP7 secretion is not detected in the MCF7 population [48]. Furthermore, in contrast to stable difference in the ΔΨm of SW620-derived subclones [11, 20], the intrinsic ΔΨm of neither SW480- nor MCF7-derived subclones is significantly associated with levels of MMP7 secretion (Figure 5(b), *P* = 0.303 and not shown, resp.).

Because acquisition of an invasive phenotype in colonic carcinoma is linked to upregulation of the actin binding protein fascin [49, 50], we next determined fascin protein levels in the parental cell populations and in subcloned cell lines by immunoblotting (Figure 5(c)). Consistent with the role of fascin in invasion, there is a significant association between fascin and the intrinsic ΔΨm of subcloned cell lines derived from the SW620 metastatic colonic carcinoma cell line (*P* = 0.007), with elevated ΔΨm linked to higher levels of fascin protein, similar to the association between elevated ΔΨm of SW620 subclones and MMP7 secretion [11, 20]. In contrast, however, fascin levels are *not *correlated with the intrinsic ΔΨm of subclones derived from primary colon tumor cell line SW480 (*P* = 0.539) nor in the subclones derived from MCF7 mammary carcinoma cells (*P* = 0.415; not shown).

### 3.5. Unlike Subclones Derived from Metastatic SW620 Colonic Tumor Cells, Differences in the Intrinsic ΔΨm of Subcloned Cell Lines Derived from Paired Primary SW480 Colon Tumor Cells Are Not Linked to Differences in Invasion

**Figure 6:**
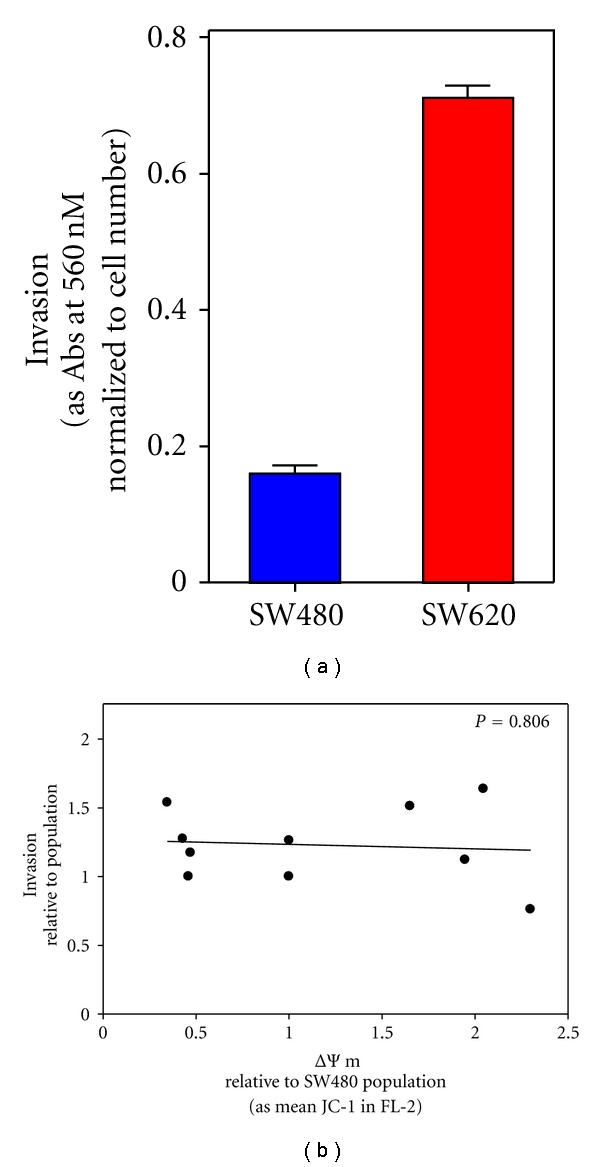
Differences in the intrinsic ΔΨm of subcloned cell lines derived from primary colon tumor are not linked to differences in invasion. SW480 and SW620 cells (a) and subcloned cell lines derived from SW480 cells (b) were seeded into chambers containing a reconstituted basement membrane overlying a polycarbonate membrane. Invasion was quantified by staining cells adhering to the polycarbonate and determining optical density (560 nM) normalized to cells/chamber. Invasion of parental SW480 and derived subclones are plotted as a function of relative ΔΨm (b).

Finally, because we have shown that the intrinsic ΔΨm of subcloned cell lines derived from metastatic SW620 cells correlates with their ability to invade an artificial basement membrane *in vitro* [11, 20], we investigated the influence of differences in the intrinsic ΔΨm on invasive capacity of subclones derived from SW480 cells, established from the primary tumor of the same patient. Consistent with previous reports [46, 47], SW480 cells exhibited approximately 77% lower invasion through reconstituted basement membrane when compared to SW620 cells (Figure 6(a); *P* < 0.0001). Furthermore, as suggested by the absence of an association between the intrinsic ΔΨm and MMP7 secretion or fascin expression levels (Figures 5(b) and 5(c), resp.), differences in the intrinsic ΔΨm of subcloned cell lines derived from SW480 primary colonic tumor cells are *not* associated with coincident differences ability to invade a reconstituted basement membrane (Figure 6(b); *P* = 0.806). 

## 4. Discussion

Heterogeneity is a fundamental property of cellular systems, including solid tumors [1]. Diversity among tumor cells likely provides reservoirs of cells that can tolerate and/or rapidly respond to changing environmental conditions, and/or escape preventative or therapeutic intervention, thereby increasing the probability of tumor expansion and progression in spite of shifting microenvironments. Consistent with this, the data presented here, combined with our previous work [11], establish that there exist subpopulations of cells with stable differences in the intrinsic ΔΨm in paired primary and metastatic colonic carcinoma cell lines (SW480 and SW620, resp.) as well as in a primary mammary carcinoma cell line (MCF7) and demonstrate the impact of intrinsic ΔΨm on tumor cell phenotype.

We show that in subclones derived from each of these cell lines, differences in intrinsic ΔΨm are linked to sensitivity to the chemoprotective agent NaB, with increased ΔΨm linked to decreased NaB-mediated cytotoxicity. Because malignant transformation is associated with a general elevation in the ΔΨm [5–9], these data suggest that the normally chemoprotective effects of NaB may be particularly diminished in subpopulations of cells with relatively higher intrinsic ΔΨm, thereby increasing the probability of their contribution to tumor progression. Moreover, we have reported previously that, unlike NaB, derivatives that are inefficiently metabolized by mitochondrial *β*-oxidation, including branched isobutyric acid and a fluorine-substituted analogue, heptafluorobutyric acid [51], are ineffective in inducing the same responses that are mediated by NaB in colonic carcinoma cells *in vitro* [25, 52, 53]. Therefore, it is likely that the metabolism of NaB plays a critical role is its effects on tumor cells *in vitro* and that the differences in NaB sensitivity associated with intrinsic ΔΨm of subcloned cell lines derived from metastatic and primary colon tumors, and from a primary mammary tumor, may be related to alterations in mitochondrial metabolic activity. 

We also show that differences in intrinsic ΔΨm in subclones derived from primary mammary carcinoma cells, as well as primary and metastatic colon carcinoma cells, are linked to phenotypes consistent with expansion of solid tumors with elevated intrinsic ΔΨm associated with increased levels of constitutive, hypoxia-independent VEGF secretion. These results suggest the presence of pseudohypoxia, the activation of hypoxia-like pathway(s) under normoxic conditions, in cells with elevated intrinsic ΔΨm. Pseudohypoxia is achieved by either defective TCA cycle-mediated impaired degradation of HIF1*α* or by accelerated mTOR-mediated translation of HIF1*α* [54–57]. Activation of the mTOR pathway is sensitive to mitochondrial function and the ΔΨm [58, 59] and our data suggest increased constitutive mTOR activation in subcloned cell lines with elevated intrinsic ΔΨm (Heerdt and Houston, in preparation).

Whereas stable differences in the intrinsic ΔΨm of subclones derived from the metastatic colonic carcinoma cell line are linked to differences in invasive capacity [11], differences in intrinsic ΔΨm are *not* linked to invasive potential in subclones derived from the paired primary colonic carcinoma cell line or from the primary mammary tumor cell line investigated here. Therefore, while subpopulations of cells with higher intrinsic ΔΨm from both primary and metastatic colonic tumors, and from a primary mammary tumor, acquire properties likely reflecting endogenous cellular resilience, demonstrated by decreased sensitivity to NaB-induced cytotoxicity, or are primed to rapidly adjust to alterations in microenvironment, demonstrated by constitutive hypoxia-independent VEGF secretion, other properties, such as enhanced invasive potential, appear only in subpopulations of cells with elevated ΔΨm that are components of a metastatic tumor. Thus, the relationship between the intrinsic ΔΨm and invasive potential may be secondary to its relationship with phenotypes associated with local solid tumor expansion. 

Although the mechanisms involved in generating and maintaining differences in ΔΨm are unclear, they may reflect alterations in the composition of mitochondrial membranes [60–65] (Mariadason and Heerdt, unpublished observation), modulations in expression of mitochondrial targeted nuclear genes [66], or enrichment in a particular mitochondrial population. As many as 90% of colorectal tumors have at least one somatic point mutation in mitochondrial DNA (mtDNA) [67–70], the majority of which are heteroplasmic, in that both wild-type and mutant mitochondrial genomes are present in the tumor [67–70]. Because the mt genome encodes subunit components of the electron transport chain, which is responsible for generating the ΔΨm, mtDNA mutations, can impact the ΔΨm [4, 71–74]. Moreover, there is at least one common heteroplasmic mtDNA mutation in subcloned cell lines with elevated intrinsic ΔΨm derived from either the SW620 or SW480 cell lines, which was* not* identified in cells with decreased ΔΨm (Heerdt et al., unpublished observation). There may also be additional heteroplasmic mtDNA populations in SW620 metastatic compared to SW480 primary colonic carcinoma cell lines [75]. Therefore, accumulation and/or enrichment of particular mtDNA mutations may reflect and/or generate stable differences in intrinsic ΔΨm and accompanying tumorigenic phenotype, and that added mtDNA alterations may be further linked to increasing clinical stage.

Interestingly, in patients with stage III colon cancers, resistance to fluorouracil-based chemotherapy has been linked to specific somatic mtDNA mutations [76], and we have found a significant correlation between the ΔΨm of colonic carcinoma cell lines and sensitivity to 5-fluorouracil (Heerdt et al., unpublished observation). Thus, alterations in intrinsic ΔΨm, perhaps associated with accumulation and/or enrichment of particular mtDNA populations, impact cellular response to chemoprotective, as well as to chemotherapeutic, agents.

The steep electrochemical proton gradient across the mitochondrial inner membrane is mainly accounted for by its matrix-side-negative electrical component, the ΔΨm. Based on Nernst's law, cations capable of permeating biological membranes can accumulate in the mitochondrial matrix at a 10-fold higher level with each 60 mV of transmembrane voltage [77]. Therefore, although the mechanisms generating and maintaining the stable relative differences in ΔΨm remain to be established, modest elevations in the ΔΨm can be exploited by mitochondriotropic agents to preferentially target attached cytotoxic agents into the mitochondrial matrix [6, 9, 76, 78, 79], particularly of cells within both primary and metastatic tumor populations that our data show as the most likely to contribute to tumor expansion and progression. 

In summary, our data suggest that stable differences in intrinsic ΔΨm among cells that comprise colon and mammary tumors reflect cellular heterogeneity and identify cells that are important in tumor progression and potential predictors of response to, and targets of, chemoprotection and chemotherapy.

## References

[1] Altschuler SJ, Wu LF (2010). Cellular heterogeneity: do differences make a difference?. *Cell*.

[2] Modica-Napolitano JS, Steele GD, Chen LB (1989). Aberrant mitochondria in two human colon carcinoma cell lines. *Cancer Research*.

[3] Sun AS, Sepkowitz K, Geller SA (1981). A study of some mitochondrial and peroxisomal enzymes in human colonic adenocarcinoma. *Laboratory Investigation*.

[4] Kulawiec M, Owens KM, Singh KK (2009). MtDNA G10398A variant in African-American women with breast cancer provides resistance to apoptosis and promotes metastasis in mice. *Journal of Human Genetics*.

[5] Summerhayes IC, Lampidis TJ, Bernal SD (1982). Unusual retention of rhodamine 123 by mitochondria in muscle and carcinoma cells. *Proceedings of the National Academy of Sciences of the United States of America*.

[6] Chen LB (1988). Mitochondrial membrane potential in living cells. *Annual Review of Cell Biology*.

[7] Chen LB, Rivers EN, Carney D, Sikora K (1990). Mitochondria in cancer cells. *Genes and Cancer*.

[8] Wong JR, Chen LB (1988). Recent advances in the study of mitochondria in living cells. *Advances in Molecular and Cell Biology*.

[9] Davis S, Weiss MJ, Wong JR (1985). Mitochondrial and plasma membrane potentials cause unusual accumulation and retention of rhodamine 123 by human breast adenocarcinoma-derived MCF-7 cells. *Journal of Biological Chemistry*.

[10] Leibovitz A, Stinson JC, McCombs WB, McCoy CE, Mazur KC, Mabry ND (1976). Classification of human colorectal adenocarcinoma cell lines. *Cancer Research*.

[11] Heerdt BG, Houston MA, Augenlicht LH (2005). The intrinsic mitochondrial membrane potential of colonic carcinoma cells is linked to the probability of tumor progression. *Cancer Research*.

[12] Soule HD, Vazquez J, Long A (1973). A human cell line from a pleural effusion derived from a breast carcinoma. *Journal of the National Cancer Institute*.

[13] Schöllnberger H, Beerenwinkel N, Hoogenveen R, Vineis P (2010). Cell selection as driving force in lung and colon carcinogenesis. *Cancer Research*.

[14] Heerdt BG, Houston MA, Wilson AJ, Augenlicht LH (2003). The intrinsic mitochondrial membrane potential (ΔΨm) is associated with steady-state mitochondrial activity and the extent to which colonic epithelial cells undergo butyrate-mediated growth arrest and apoptosis. *Cancer Research*.

[15] Heerdt BG, Houston MA, Mariadason JM, Augenlicht LH (2000). Dissociation of staurosporine-induced apoptosis from G2-M arrest in SW620 human colonic carcinoma cells: initiation of the apoptotic cascade is associated with elevation of the mitochondrial membrane potential (ΔΨm). *Cancer Research*.

[16] Heerdt BG, Houston MA, Anthony GM, Augenlicht LH (1999). Initiation of growth arrest and apoptosis of MCF-7 mammary carcinoma cells by tributyrin, a triglyceride analogue of the short-chain fatty acid butyrate, is associated with mitochondrial activity. *Cancer Research*.

[17] Heerdt BG, Houston MA, Anthony GM, Augenlicht LH (1998). Mitochondrial membrane potential (ΔΨmt) in the coordination of p53-independent proliferation and apoptosis pathways in human colonic carcinoma cells. *Cancer Research*.

[18] Mosmann T (1983). Rapid colorimetric assay for cellular growth and survival: application to proliferation and cytotoxicity assays. *Journal of Immunological Methods*.

[19] Reers M (1991). J-aggregate formation of a carbocyanine as a quantitative fluorescent indicator of membrane potential. *Biochemistry*.

[20] Heerdt BG, Houston MA, Augenlicht LH (2006). Growth properties of colonic tumor cells are a function of the intrinsic mitochondrial membrane potential. *Cancer Research*.

[21] Roediger WEW (1980). Role of anaerobic bacteria in the metabolic welfare of the colonic mucosa in man. *Gut*.

[22] Cummings JH (1983). Fermentation in the human large intestine: evidence and implications for health. *The Lancet*.

[23] Schulz H, Vance DE, Vance JE (1985). Oxidation of fatty acids. *Biochemistry of Lipids and Membranes*.

[24] Roediger WEW (1982). Utilization of nutrients by isolated epithelial cells of the rat colon. *Gastroenterology*.

[25] Heerdt BG, Houston MA, Augenlicht LH (1994). Potentiation by specific short-chain fatty acids of differentiation and apoptosis in human colonic carcinoma cell lines. *Cancer Research*.

[26] Roediger WEW (1988). Bacterial short-chain fatty acids and mucosal diseases of the colon. *British Journal of Surgery*.

[27] Augenlicht LH, Anthony GM, Church TL (1999). Short-chain fatty acid metabolism, apoptosis, and Apc-initiated tumorigenesis in the mouse gastrointestinal mucosa. *Cancer Research*.

[28] Mariadason JM, Corner GA, Augenlicht LH (2000). Genetic reprogramming in pathways of colonic cell maturation induced by short chain fatty acids: comparison with trichostatin A, sulindac, and curcumin and implications for chemoprevention of colon cancer. *Cancer Research*.

[29] Medina V, Afonso JJ, Alvarez-Argijelles H, Hernandez C, Gonzalez F (1998). Sodium butyrate inhibits carcinoma development in a 1,2-dimethylhydrazine-induced rat colon cancer. *Journal of Parenteral and Enteral Nutrition*.

[30] D’Argenio G, Cosenza V, Delle Cave M (1996). Butyrate enemas in experimental colitis and protection against large bowel cancer in a rat model. *Gastroenterology*.

[31] McIntyre A, Gibson PR, Young GP (1993). Butyrate production from dietary fibre and protection against large bowel cancer in a rat model. *Gut*.

[32] Planchon P, Raux H, Magnien V (1991). New stable butyrate derivatives alter proliferation and differentiation in human mammary cells. *International Journal of Cancer*.

[33] Mandal M, Kumar R (1996). Bcl-2 expression regulates sodium butyrate-induced apoptosis in human MCF-7 breast cancer cells. *Cell Growth and Differentiation*.

[34] Novagrodsky A, Ovir A, Ravid A (1983). Effect of polar organic compounds in leukemia cells. Butyrate-induced partial remission of acute myalogenous leukemia in a child. *Cancer*.

[35] Miller AA, Kurschel E, Osieka R, Schmidt CG (1987). Clinical pharmacology of sodium butyrate in patients with acute leukemia. *European Journal of Cancer and Clinical Oncology*.

[36] Newmark HL, Lupton JR, Young CW (1994). Butyrate as a differentiating agent: pharmacokinetics, analogues and current status. *Cancer Letters*.

[37] Yuan Z, Eiseman J, Plasance K (1994). Plasma pharmacoinetics of butyrate after the administration of tributyrin and Na butyrate to mice and rats. *Proceedings of the American Association for Cancer Research*.

[38] Cummings JH, Pomare EW, Branch WJ, Naylor CPE, Macfarlane GT (1987). Short chain fatty acids in human large intestine, portal, hepatic and venous blood. *Gut*.

[39] Ferrara N (2004). Vascular endothelial growth factor: basic science and clinical progress. *Endocrine Reviews*.

[40] Bergers G, Benjamin LE (2003). Tumorigenesis and the angiogenic switch. *Nature Reviews Cancer*.

[41] Hicklin DJ, Ellis LM (2005). Role of the vascular endothelial growth factor pathway in tumor growth and angiogenesis. *Journal of Clinical Oncology*.

[42] Tokunaga T, Oshika Y, Abe Y (1998). Vascular endothelial growth factor (VEGF) mRNA isoform expression pattern is correlated with liver metastasis and poor prognosis in colon cancer. *British Journal of Cancer*.

[43] Kioi M, Yamamoto K, Higashi S, Koshikawa N, Fujita K, Miyazaki K (2003). Matrilysin (MMP-7) induces homotypic adhesion of human colon cancer cells and enhances their metastatic potential in nude mouse model. *Oncogene*.

[44] Ichikawa Y, Ishikawa T, Momiyama N (1998). Detection of regional lymph node metastases in colon cancer by using RT-PCR for matrix metalloproteinase 7, matrilysin. *Clinical and Experimental Metastasis*.

[45] Mori M, Barnard GF, Mimori K, Ueo H, Akiyoshi T, Sugimachi K (1995). Overexpression of matrix metalloproteinase-7 mRNA in human colon carcinomas. *Cancer*.

[46] Witty JP, McDonnell S, Newell KJ (1994). Modulation of matrilysin levels in colon carcinoma cell lines affects tumorigenicity in vivo. *Cancer Research*.

[47] Ho BY, Wu YM, Hsu YW (2010). Effects of monascus-fermented rice extract on malignant cell-associated neovascularization and intravasation determined using the chicken embryo chorioallantoic membrane model. *Integrative Cancer Therapies*.

[48] Wang F, Reierstad S, Fishman DA (2006). Matrilysin over-expression in MCF-7 cells enhances cellular invasiveness and pro-gelatinase activation. *Cancer Letters*.

[49] Jawhari AU, Buda A, Jenkins M (2003). Fascin, an actin-bundling protein, modulates colonic epithelial cell invasiveness and differentiation in vitro. *American Journal of Pathology*.

[50] Hashimoto Y, Skacel M, Lavery IC, Mukherjee AL, Casey G, Adams JC (2006). Prognostic significance of fascin expression in advanced colorectal cancer: an immunohistochemical study of colorectal adenomas and adenocarcinomas. *BMC Cancer*.

[51] Goldman P (1969). The carbon-fluorine bond in compounds of biological interest. *Science*.

[52] Heerdt BG, Augenlicht LH (1991). Effects of fatty acids on expression of genes encoding subunits of cytochrome c oxidase and cytochrome c oxidase activity in HT29 human colonic adenocarcinoma cells. *Journal of Biological Chemistry*.

[53] Bordonaro M, Mariadason JM, Aslam F, Heerdt BG, Augenlicht LH (1999). Butyrate-induced apoptotic cascade in colonic carcinoma cells: modulation of the *β*-catenin-Tcf pathway and concordance with effects of sulindac and trichostatin A but not curcumin. *Cell Growth and Differentiation*.

[54] King A, Selak MA, Gottlieb E (2006). Succinate dehydrogenase and fumarate hydratase: linking mitochondrial dysfunction and cancer. *Oncogene*.

[55] Koivunen P, Hirsilä M, Remes AM, Hassinen IE, Kivirikko KI, Myllyharju J (2007). Inhibition of hypoxia-inducible factor (HIF) hydroxylases by citric acid cycle intermediates: possible links between cell metabolism and stabilization of HIF. *Journal of Biological Chemistry*.

[56] Pollard PJ, Brière JJ, Alam NA (2005). Accumulation of Krebs cycle intermediates and over-expression of HIF1*α* in tumours which result from germline FH and SDH mutations. *Human Molecular Genetics*.

[57] Selak MA, Armour SM, MacKenzie ED (2005). Succinate links TCA cycle dysfunction to oncogenesis by inhibiting HIF-*α* prolyl hydroxylase. *Cancer Cell*.

[58] Schieke SM, Phillips D, McCoy JP (2006). The mammalian target of rapamycin (mTOR) pathway regulates mitochondrial oxygen consumption and oxidative capacity. *Journal of Biological Chemistry*.

[59] Kim DH, Sarbassov DD, Ali SM (2002). mTOR interacts with raptor to form a nutrient-sensitive complex that signals to the cell growth machinery. *Cell*.

[60] Jiang F, Ryan MT, Schlame M (2000). Absence of cardiolipin in the crd1 null mutant results in decreased mitochondrial membrane potential and reduced mitochondrial function. *Journal of Biological Chemistry*.

[61] Hong MY, Chapkin RS, Barhoumi R (2002). Fish oil increases mitochondrial phospholipid unsaturation, upregulating reactive oxygen species and apoptosis in rat colonocytes. *Carcinogenesis*.

[62] Vaz FM, Houtkooper RH, Valianpour F, Barth PG, Wanders RJA (2003). Only one splice variant of the human TAZ gene encodes a functional protein with a role in cardiolipin metabolism. *Journal of Biological Chemistry*.

[63] Barth PG, Wanders RJA, Vreken P (1999). X-linked cardioskeletal myopathy and neutropenia (Barth syndrome)-MIM 302060. *Journal of Pediatrics*.

[64] Schlame M, Kelley RI, Feigenbaum A (2003). Phospholipid abnormalities in children with Barth syndrome. *Journal of the American College of Cardiology*.

[65] Xu Y, Kelley RI, Blanck TJJ, Schlame M (2003). Remodeling of cardiolipin by phospholipid transacylation. *Journal of Biological Chemistry*.

[66] Lu H, Cao X (2008). GRIM-19 is essential for maintenance of mitochondrial membrane potential. *Molecular Biology of the Cell*.

[67] He Y, Wu J, Dressman DC (2010). Heteroplasmic mitochondrial DNA mutations in normal and tumour cells. *Nature*.

[68] Polyak K, Li Y, Zhu H (1998). Somatic mutations of the mitochondrial genome in human colorectal tumours. *Nature Genetics*.

[69] Habano W, Nakamura SI, Sugai T (1998). Microsatellite instability in the mitochondrial DNA of colorectal carcinomas: evidence for mismatch repair systems in mitochondrial genome. *Oncogene*.

[70] Habano W, Sugai T, Nakamura S, Uesugi N, Yoshida T, Sasou S (2000). Microsatellite instability and mutation of mitochondrial and nuclear DNA in gastric carcinoma. *Gastroenterology*.

[71] Kulawiec M, Owens KM, Singh KK (2009). Cancer cell mitochondria confer apoptosis resistance and promote metastasis. *Cancer Biology &amp; Therapy*.

[72] Munakata K, Tanaka M, Mori K (2004). Mitochondrial DNA 3644T→C mutation associated with bipolar disorder. *Genomics*.

[73] Janssen RJRJ, Nijtmans LG, van den Heuvel LP, Smeitink JAM (2006). Mitochondrial complex I: structure, function and pathology. *Journal of Inherited Metabolic Disease*.

[74] Abramov AY, Smulders-Srinivasan TK, Kirby DM (2010). Mechanism of neurodegeneration of neurons with mitochondrial DNA mutations. *Brain*.

[75] Lee HC, Hsu LS, Yin PH, Lee LM, Chi CW (2007). Heteroplasmic mutation of mitochondrial DNA D-loop and 4977-bp deletion in human cancer cells during mitochondrial DNA depletion. *Mitochondrion*.

[76] Lièvre A, Chapusot C, Bouvier A-M (2005). Clinical value of mitochondrial mutations in colorectal cancer. *Journal of Clinical Oncology*.

[77] Azzone GF, Petronilli V, Zoratti M (1984). ’Cross-talk’ between redox- and ATP-driven H+ pumps. *Biochemical Society Transactions*.

[78] Fantin VR, Berardi MJ, Scorrano L, Korsmeyer SJ, Leder P (2002). A novel mitochondriotoxic small molecule that selectively inhibits tumor cell growth. *Cancer Cell*.

[79] Dorward AM, Singh G (1996). Energetic characteristics of cisplatin resistant ovarian carcinoma cells. *Anticancer Research*.

